# Decoding microglial immunometabolism: a new frontier in Alzheimer's disease research

**DOI:** 10.1186/s13024-025-00825-0

**Published:** 2025-03-27

**Authors:** Eun Sun Jung, Hayoung Choi, Inhee Mook-Jung

**Affiliations:** 1 Convergence Dementia Research Center, Seoul National University College of Medicine, Seoul, South Korea; 2 Department of Biomedical Sciences, Seoul National University College of Medicine, Seoul, South Korea; 3 Korea Dementia Research Center, Seoul, South Korea

**Keywords:** Microglia, Immunometabolism, Metabolic reprogramming, Neuroinflammation, APOE, TREM2, HIF, Hexokinase, Aβ, Tau

## Abstract

Alzheimer’s disease (AD) involves a dynamic interaction between neuroinflammation and metabolic dysregulation, where microglia play a central role. These immune cells undergo metabolic reprogramming in response to AD-related pathology, with key genes such as TREM2, APOE, and HIF-1α orchestrating these processes. Microglial metabolism adapts to environmental stimuli, shifting between oxidative phosphorylation and glycolysis. Hexokinase-2 facilitates glycolytic flux, while AMPK acts as an energy sensor, coordinating lipid and glucose metabolism. TREM2 and APOE regulate microglial lipid homeostasis, influencing Aβ clearance and immune responses. LPL and ABCA7, both associated with AD risk, modulate lipid processing and cholesterol transport, linking lipid metabolism to neurodegeneration. PPARG further supports lipid metabolism by regulating microglial inflammatory responses. Amino acid metabolism also contributes to microglial function. Indoleamine 2,3-dioxygenase controls the kynurenine pathway, producing neurotoxic metabolites linked to AD pathology. Additionally, glucose-6-phosphate dehydrogenase regulates the pentose phosphate pathway, maintaining redox balance and immune activation. Dysregulated glucose and lipid metabolism, influenced by genetic variants such as APOE4, impair microglial responses and exacerbate AD progression. Recent findings highlight the interplay between metabolic regulators like REV-ERBα, which modulates lipid metabolism and inflammation, and Syk, which influences immune responses and Aβ clearance. These insights offer promising therapeutic targets, including strategies aimed at HIF-1α modulation, which could restore microglial function depending on disease stage. By integrating metabolic, immune, and genetic factors, this review underscores the importance of microglial immunometabolism in AD. Targeting key metabolic pathways could provide novel therapeutic strategies for mitigating neuroinflammation and restoring microglial function, ultimately paving the way for innovative treatments in neurodegenerative diseases.

## Background

Alzheimer’s disease (AD) is a multifactorial and polygenic disorder resulting from complex interactions between various genetic and environmental factors [1]. The clinical heterogeneity of AD, characterized by diverse symptoms among patients [2–4], complicates therapeutic strategies. Additionally, AD is closely associated with metabolic syndrome, which includes hypertension, hyperlipidemia, obesity, and type 2 diabetes mellitus (T2DM) [5–8]. Given this complexity, the efficacy of monotherapies, such as Aβ-targeting antibody treatments, remains limited, highlighting the growing need for combination therapy strategies [9–11].

Consistent with this perspective, genome-wide association studies (GWAS) have identified numerous genetic risk factors for late-onset Alzheimer’s disease (LOAD), with a significant proportion being linked to microglial function, underscoring their central role in disease pathology [12–14]. These risk genes are involved in essential microglial processes, including Aβ clearance, lipid metabolism, and inflammatory regulation, highlighting microglial dysfunction as a key driver of AD pathogenesis [14–16].

As the resident immune cells of the central nervous system (CNS), microglia play a crucial role in innate immune responses by rapidly detecting and responding to pathogen-associated molecular patterns (PAMPs) and damage-associated molecular patterns (DAMPs) [17–21]. Upon activation, microglia produce pro-inflammatory cytokines, and engage in phagocytic activity, making them key regulators of the pathological environment [22]. Consequently, their ability to clear Aβ and modulate neuroinflammation is critical in AD progression, highlighting the regulation of neuroinflammatory pathways as a promising therapeutic approach for AD [23–26].

Importantly, microglial function is closely linked to metabolic processes. Microglial plasticity—the ability to functionally and phenotypically adapt to environmental stimuli [27, 28]—is tightly regulated by metabolic flexibility [29, 30]. This metabolic adaptability enables microglia to efficiently utilize glucose, amino acids, and lipids to sustain energy-intensive processes, such as pro-inflammatory cytokine production and phagocytosis, under both homeostatic and pathological conditions [29, 31–33].

Among the risk genes identified through GWAS, *TREM2* and *APOE* stand out as key regulators of multiple metabolic pathways, including glucose, lipid, and amino acid metabolism [34–37]. By influencing microglial energy balance, immune responses, and phagocytic activity, these genes further underscore their complex association with AD pathology [38–42].

**Fig. 1:**
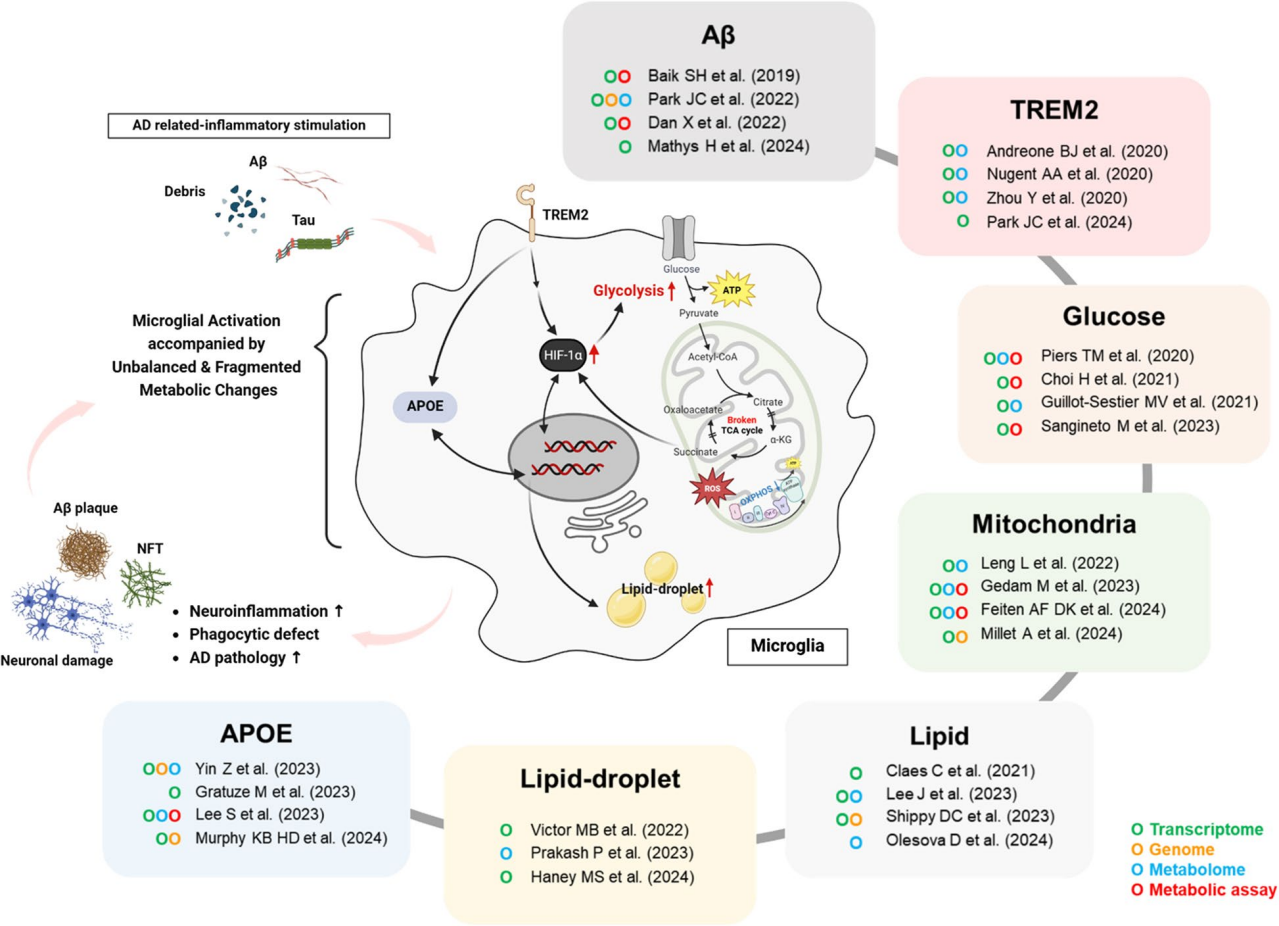
Major metabolic alterations and regulatory factors in AD microglia revealed by immuno-metabolomics approaches. Microglia function and phenotype change dynamically as AD pathogenesis progresses, accompanied by significant metabolic alterations. Initially, these cells demonstrate metabolic flexibility, but as the disease becomes chronic, microglia gradually lose their adaptive capacity. This reliance on biased and fragmented metabolic pathways eventually leads to insufficient energy and material supply, resulting in functional impairment and accelerated disease progression. The first notable metabolic shift in AD microglia involves glucose metabolism, characterized by excessive glucose uptake and increased dependence on non-aerobic glycolysis via HIF-1α pathway. This change subsequently leads to the inhibition and breakdown of mitochondrial energy metabolism. Furthermore, prolonged exposure to Aβ plaques, neurofibrillary tangles (NFTs), and excessive cell debris dramatically alters overall lipid metabolism, resulting in the accumulation of lipid droplets (LDs). Microglia with excessive LD accumulation, known as lipid-droplet-accumulating microglia (LDAMs), exhibit significantly reduced phagocytic ability and enhanced inflammatory properties. Recent studies have highlighted the crucial roles of APOE and TREM2, both high-risk genes for AD, in mediating these metabolic transitions in microglia. These genes are increasingly recognized as key players in modulating microglial function and metabolism in the context of AD. The advent of multi-omics approaches has accelerated the identification of candidate substances involved in microglial function and metabolic regulation pathways. This comprehensive analysis provides a deeper understanding of the complex interplay between immune response and metabolism in AD microglia

Furthermore, recent multi-omics studies have revealed that distinct microglial phenotypes are strongly associated with specific metabolic pathways in the context of neurodegenerative diseases such as AD (Fig. 1, Table 1). These findings highlight the crucial role of microglial metabolic regulation in AD pathogenesis and suggest that targeting these pathways may provide novel therapeutic opportunities [43–45].

**Table 1:** Summary of AD microglial omics and metabolism studies

Models		Sample types	Modulation	Key Findings	Screening Methods	References
**AD animal model microglia**	APP/PS1 (M,F; 16-18 M)	isolated adult mouse microglia		↑ Glycolysis in females vs. males	multiplexed gene expression analysis; LC/MS	Guillot-Sestier et al. (2021) [90]
	APP/PS1	isolated adult mouse microglia	TREM2 depletion	TREM2-dependent changes in OXPHOS & cholesterol metabolism	bulk RNA-seq; metabolomics; lipidomics	Feiten et al. (2024) [248]
	5XFAD (M,F; 3-7 M)	isolated adult mouse microglia		↑ LD accumulation, ↓ FFAs, ↑ TGs	Lipidomics	Prakash et al. (2024) [66]
	5XFAD (-; 2,6,9 M)	hippocampus		Altered glucose metabolism (transporters, glycolysis, OXPHOS)	scRNA-seq	Choi et al. (2021) [127]
	5XFAD (F; 9 M)	brain		↑ Glycolysis, mitochondrial repurposing, ↑ proton leak	scRNA-seq	Sangineto et al. (2023) [92]
	5XFAD (F; 7.5 M)	isolated adult mouse microglia	oAβ ICV injection	Aβ-induced acute inflammation & tolerance depend on glycolysis shift	bulk RNA-seq	Baik et al. (2019) [46]
	5XFAD (-; 12 M)	isolated adult mouse microglia	PKM2 inhibition	Glycolysis/H4K12la/PKM2 positive feedback loop exacerbates microglial dysfunction	genome-wide CUT&Tag analysis	Pan et al. (2022) [114]
	5XFAD (M,F; 1,3,6,8 M)	isolated adult mouse microglia	TREM2 depletion	TREM2 required for full DAM activation (↑ phagocytosis & lipid metabolism)	scRNA-seq; ChiP	Keren-Shaul et al. (2017) [157]
	5XFAD (F; 8)	isolated adult mouse microglia, BMDM	TREM2 depletion	TREM2 loss → AMPK-driven autophagy, ↓ anabolic & energetic metabolism	Microarray; EIS-MS/MS	Ulland et al. (2017) [35]
	5XFAD (M,F; 7,15 M)	brain	TREM2 depletion	↓ Microglia-expressed protein abundance in Trem2–/–	snRNA-seq; proteomics	Zhou et al. (2020) [253]
	5XFADiPKR; 5XFAD*Eif*2^A^ (M, F; 6 M); PS19iPKR; PS19Eif2A (M,F; 8 M)	isolated adult mouse microglia; Microglia-specific ribosomal profiling (TRAP)	microglia specific ISR modulation	Glycolytic & lipid metabolism changes, toxic lipid secretion	snRNA-seq; TRAP seq	Flurry et al. (2024) [196]
	5XFAD; hAPOE (-; 2,24 M)	brain	human APOE2, APOE3, APOE4 KI	TIM depletion in cellular energetics (TCA cycle, glycolysis, PPP); ↑ ROS detox pathways & amino acid metabolism	scRNA-seq/scATAC-seq multiome; interactome	Millet et al. (2024) [259]
	hAPOE KI (F; 3,12,24 M)	region-specific	human APOE3, APOE4 KI	APOE4 microglia: ↑ Aerobic glycolysis, ↑ Hif1α, altered lipid metabolism	snRNA-seq; spatial transcriptomics; metabolomics	Lee et al. (2023) [70]
	hAPOE KI; P301S:hAPOE KI; APP/PS1:hAPOE KI	brain; isolated adult mouse microglia	human APOE3, APOE4 KI and conditional KO; LGALS3 stereotaxic injection; Inpp5d modulation; ITGB8 inhibition	APOE4-ITGB8-TGFβ as a negative regulator of MGnD response; altered lipid species	scRNA-seq; ChIP-seq; lipidomics	Yin et al. (2023) [256]
	P301S:hAPOE KI; P301S:APOE KO (male; 9 M)	hippocampus; BMDM	TREM2 depletion	ApoE4 & tau-associated microgliosis → TREM2-independent lysosomal lipid accumulation	snRNA-seq (FANS)	Gratuze et al. (2023) [274]
	APP NL-G-F (M,F; 9 M)	isolated adult mouse microglia; primary microglia	C3aRKO	C3aR + microglia: dysfunctional metabolism; C3aR1-null microglia resistant to hypoxia-induced lipid accumulation; C3aR ablation rescued lipid profiles & improved phagocytosis	bulk RNA-seq	Gedam et al. (2023) [309]
	APP^SAA^ (M,F; 5-20 M)	isolated adult mouse microglia; brain biofluid		Microglia with high intracellular Aβ → significant lipid & metabolite changes	bulk RNA-seq; LC/MS	Xia et al. (2022) [48]
	SHR24 (-; 4,6,8,10,12,14 M)	brain, CSF, plasma	Pathological tau	↑ lipid production (protein fibrillization, membrane reorganization, inflammation); ↑ phospholipids, sphingolipids, & LD accumulation in microglia	Lipidomics	Olesova et al. (2024) [47]
**Human iPSC-derived microglia**	iPSC-derived microglia	iPSC-derived microglia	TREM2-R47H variants	↓ OXPHOS & glycolysis; fails to switch to glycolysis after immune stimulation	Microarray; proteom array	Piers et al. (2020) [250]
	iPSC-derived microglia		PSEN1ΔE9, APPswe, and APOE4	APOE4 → defective glycolytic & mitochondrial metabolism	bulk RNA-seq	Konttinen et al. (2019) [257]
	iPSC-derived microglia	APOE3, APOE4; LD-high, LD-low	fAβ	↑ LD accumulation, exacerbated by APOE4; LD spectra overlap with unsaturated TGs	bulk RNA-seq; ATAC-seq lipidomics	Haney et al. (2024) [260]
	iPSC-derived microglia and neuron (spheroid)	APOE3, APOE4	neuronal conditioned medium	APOE4 → impaired lipid catabolism & lipid accumulation	bulk RNA-seq	Victor et al. (2022) [73]
	iPSC-derived microglia	APOE2, APOE3, APOE4, APOE KO	LDLR, CE	Altered cholesterol metabolism & lipid peroxidation	LCMS; RNA-seq	Guo et al. (2025) [283]
**Human postmortem brain**	AD-APOE4/4; AD-APOE3/3	frontal cortex		↑ LD accumulation, exacerbated by APOE4	snRNA-seq	Haney et al. (2024) [260]
	TREM2 variant carriers (R47H, R62H)	prefrontal cortex		TREM2-R47H → ↑ oxidative stress & lipid metabolism genes	snRNA-seq	Zhou et al. (2020) [253]
	AD-E4 patients, TREM2 variant carriers (R47H, R62H)	parietal cortex		APOE4 & tau → TREM2-independent lysosomal lipid accumulation	snRNA-seq	Gratuze et al. (2023) [274]
	AD-APOE3/4; AD-APOE3/3	brain		APOE4-ITGB8-TGFβ pathway inhibits MGnD response; lipid species alterations	bulk RNA-seq	Yin et al. (2023) [256]
	ROSMAP	region-specific		↑ Glycogen-related gene expression → glial metabolic response may not be globally coordinated	snRNA-seq	Mathys et al. (2024) [357]
	AD-E3, E4 patients;	frozen superior frontal gyrus and fusiform gyrus		↑ Lipid accumulation	snRNA-seq	Haney et al. (2024) [260]
**Xenotransplantation**	5X-hCSF1 (F, 7 M)	TREM2-R47H xenografted microglia	iPSC-derived TREM2-R47H microglia transplantation	TREM2-R47H xMGs → ↓ LD accumulation & ↓ lipid metabolism genes (SPP1, APOE, CTSD)	scRNA-seq	Claes et al. (2021) [158]
	APPNL-G-F;hCSF1	isolated microglia	iPSC-derived isogenic APOE microglia transplantation	Dysregulated lipid metabolism; ↓ CHCHD2 (mitochondrial migration gene)	bulk RNA-seq; ATAC-seq	Murphy et al. (2024) [261]

*Abbreviations*: *Aβ* amyloid beta, *AD* Alzheimer’s disease, *APOE* apolipoprotein E, *BMDM* bone marrow-derived macrophages, *CE* cholesteryl ester, *C3aR* complement component 3a receptor, *DAM* disease-associated microglia, *FFA* free fatty acids, *fAβ* fibrillar amyloid beta, *ICV* intracerebroventricular, *ISR* integrated stress response, *KI* knock-in, *LD* lipid droplet, *LDLR* low-density lipoprotein receptor, *MGnD* neurodegenerative microglia, *OXPHOS* oxidative phosphorylation, *PPP* pentose phosphate pathway, *ROS* reactive oxygen species, *SPP1* secreted phosphoprotein 1, *TCA* tricarboxylic acid cycle, *TG* triacylglycerols, *TIM* terminally inflammatory microglia, *TREM2* triggering receptor expressed on myeloid cells 2, *xMGs* xenografted microglia

This review provides a comprehensive analysis of how key metabolic pathways—glucose, lipid, and amino acid metabolism—are altered in microglia under AD conditions and their functional implications in disease progression. Each metabolic pathway is examined individually to elucidate its specific role in microglial dysfunction and its contribution to AD pathogenesis. Additionally, the impact of major AD risk genes, TREM2 and APOE, on these metabolic processes is explored, emphasizing their regulatory influence on microglial energy balance and immune responses. Lastly, key molecular mediators involved in microglial metabolism are highlighted, offering insights into the intricate regulatory mechanisms that drive metabolic adaptations in AD.

**Fig. 2:**
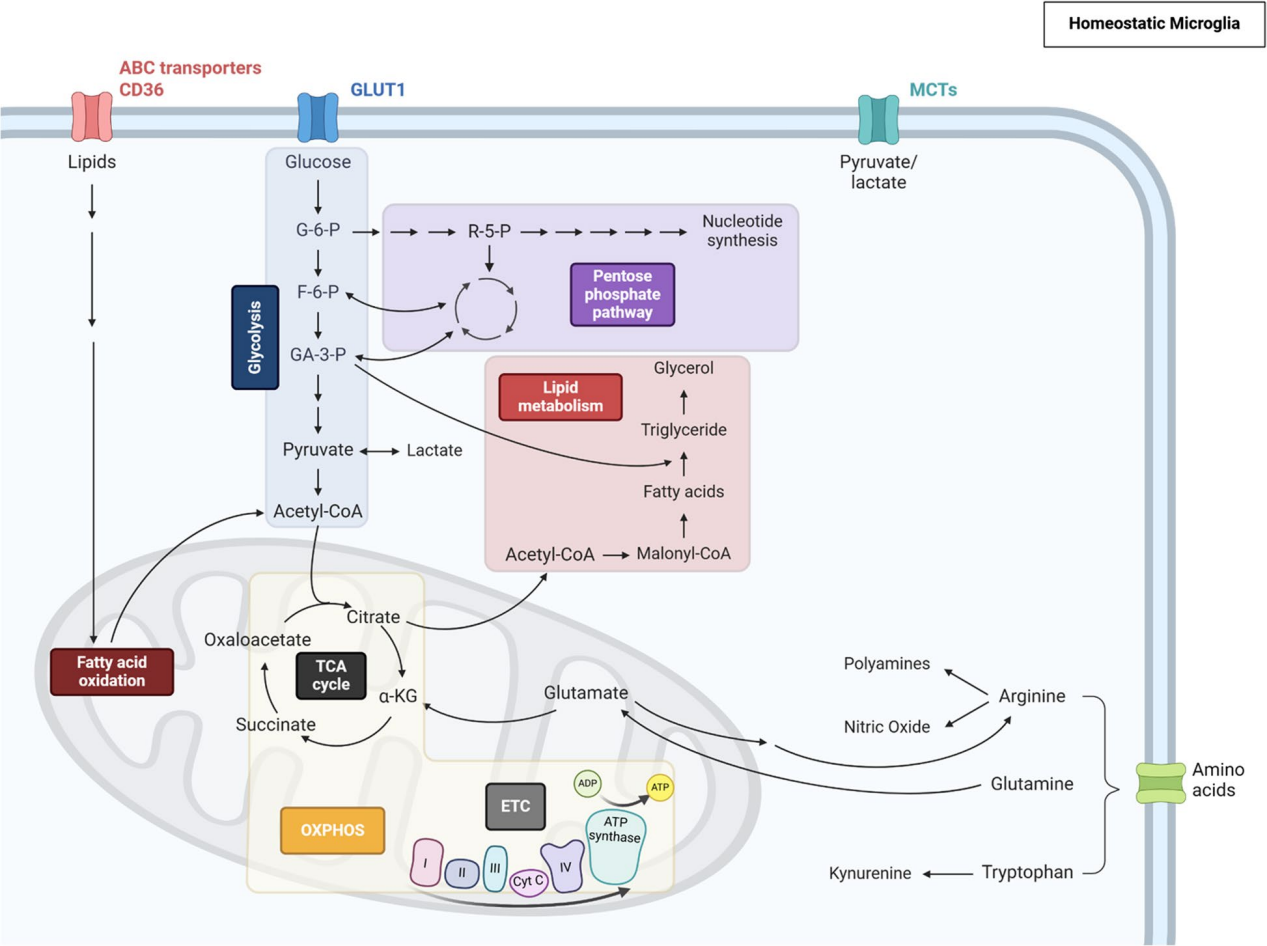
An overview of major metabolic pathways. Cellular metabolism is a complex network of interconnected pathways that respond to internal and external signals to meet the cell's needs. These pathways function in a coordinated manner to produce essential components for cellular function and are subject to regulation by various signaling mechanisms. Here is an overview of the major metabolic pathways and their roles. Glycolysis is the process by which glucose is converted into pyruvate. Subsequently, pyruvate can further transform into lactate or be integrated into the tricarboxylic acid (TCA) cycle. Within the TCA cycle, pyruvate undergoes a series of reactions that produce NADH and FADH2, which the oxidative phosphorylation (OXPHOS) electron transport chain (ETC) system uses to generate ATP. Furthermore, glycolysis supplies intermediates for the pentose phosphate pathway (PPP), which produces ribose for nucleotides and amino acids. Lipid metabolism involves synthesizing fatty acids and lipid transportation, a process involving citrate derived from the TCA cycle. Moreover, it has been demonstrated that fatty acids can undergo oxidation, generating NADH and FADH2, which, in turn, promote ATP production through the OXPHOS. Amino acid metabolism also provides vital nutrients for the TCA cycle and plays a significant role in protein biosynthesis and adequate cellular activation. The intricate interconnection and regulation of these pathways by cellular signaling ensure metabolic activity aligns with the cell's requirements. This coordinated system enables cells to produce energy and essential molecules for various physiological processes efficiently

Microglial metabolic reprogramming is not merely a response to AD pathology [46–48] but actively drives disease progression, underscoring the critical role of immunometabolism in AD [49–51]. In AD, pathological stimuli such as amyloid-beta (Aβ) and tau aggregates induce shifts in glycolysis, oxidative phosphorylation (OXPHOS), and lipid metabolism [52–54](Fig. 2). These changes are integral to microglial function, influencing their ability to clear toxic aggregates, modulate inflammation, and maintain homeostasis [49–51].

Glucose metabolism is vital for microglial function [51, 55, 56]. The brain, despite its small size, consumes about 20% of the body's energy, with glucose being the primary energy source. Microglia typically rely on oxidative phosphorylation for energy in homeostatic conditions but switch to glycolysis during inflammation, a shift that supports rapid energy production needed for immediate responses [57–59]. However, prolonged exposure to pathological stimuli such as Aβ can lead to a state of microglial immune tolerance, in which both glycolysis and OXPHOS are downregulated, impairing microglial responsiveness and contributing to AD pathology [46, 60].

Lipid metabolism also plays a crucial role in microglial function [61–64]. Aβ-driven lipid droplets (LDs) accumulation is linked to impaired phagocytosis and elevated reactive oxygen species (ROS) production, promoting neuroinflammation in AD [65, 66]. The involvement of a significant number of LOAD risk genes, including TREM2 and APOE, in lipid metabolism underscores the close relationship between microglial lipid metabolism and AD pathology [34, 37, 67].

In addition to glucose and lipids, microglia exhibit metabolic flexibility by utilizing alternative energy sources, such as amino acids, particularly in circumstances where glucose availability is limited. This metabolic adaptation enables microglia to sustain their critical functions under varying nutritional conditions [30, 68]. However, dysregulation of amino acid metabolism can exacerbate microglial activation and neuroinflammation, particularly in microglia expressing the APOE4 allele [69].

The metabolic pathways essential for microglial function are not independent but are tightly interconnected, influencing and regulating each other in a coordinated manner. TREM2 and APOE act as key regulators of microglial metabolism, orchestrating both metabolic and immune functions [39, 70]. TREM2, a receptor expressed on microglia, influences their metabolic state, lipid handling, and inflammatory responses, and its loss-of-function variants are linked to an increased risk of AD [71]. Similarly, APOE, particularly the APOE4 isoform, alters microglial metabolism by disrupting multiple metabolic pathways [72–74], leading to impaired immune responses and exacerbation of AD pathology. Furthermore, the intricate interplay between TREM2 and APOE adds another layer of complexity to the regulation of microglial metabolism and function.

Additionally, hypoxia-inducible factors (HIFs) and energy-sensing enzymes like adenosine monophosphate-activated protein kinase (AMPK) and hexokinase 2 (HK2) are crucial in regulating microglial metabolism [29, 75, 76]. HIFs drive metabolic reprogramming under hypoxic conditions [77], AMPK acts as an energy sensor to maintain cellular energy homeostasis [78], and HK2 plays a pivotal role in glucose metabolism [79]. Dysregulation of these regulatory factors, which influence multiple metabolic pathways, may contribute to the chronic neuroinflammation and metabolic dysfunction observed in AD. Thus, in the later sections of this review, we will discuss how these key regulators of metabolic processes are modulated under AD conditions.

In summary, this review provides a comprehensive overview of metabolic pathways that regulate microglial function in AD. Starting with an exploration of energy substrates like glucose, lipids, and amino acids, we then delve into molecular mediators such as TREM2, APOE, and other regulators that shape microglial immunometabolism. By integrating these findings, we aim to highlight therapeutic opportunities targeting these pathways.

## Main text

### Microglial energy substrates and metabolism in AD

#### Glucose metabolism

Glucose is the main source of energy for the cells in the CNS, including microglia [80]. These cells require significant energy expenditure to perform critical functions such as surveillance and phagocytosis, relying on ATP, the primary cellular energy molecule, produced through various metabolic processes [81, 82]. Glycolysis and mitochondrial oxidative phosphorylation (OXPHOS) are the two major metabolic pathways that provide energy for microglia [51, 82]. Glycolysis involves a series of enzyme-catalyzed reactions that convert glucose to pyruvate or lactate. Following glycolysis, pyruvate is converted into acetyl coenzyme A (acetyl CoA) to enter the tricarboxylic acid (TCA) cycle for OXPHOS [83]. Although glycolysis produces ATP less efficiently than OXPHOS, it allows for rapid ATP production without requiring oxygen [84]. Typically, homeostatic microglia primarily rely on OXPHOS for ATP production, while inflammatory microglia are thought to switch to glycolysis through metabolic reprogramming to meet their energy needs [57–59]. This shift is advantageous for microglia as it allows them to respond quickly to danger signals [81, 85], a crucial capability in conditions such as AD.

Recent evidence indicates that microglia can rapidly switch their metabolism between glycolysis and OXPHOS depending on specific conditions like those present in AD [46, 86, 87]. For example, Aβ has been shown to induce metabolic reprogramming in primary mouse microglia, shifting their preferred ATP production pathway from OXPHOS to glycolysis, a shift necessary for energy-intensive processes like phagocytosis and the production of pro-inflammatory cytokines [46, 86]. However, microglia exposed continuously to Aβ may enter a state of tolerance, marked by a significant reduction in energy metabolism, encompassing both OXPHOS and glycolysis, leading to diminished responsiveness to external stimuli [46, 86]. This tolerance phase, characterized by reduced cytokine secretion and impaired phagocytosis, may contribute to the accumulation of Aβ and the progression of AD pathology. Indeed, these characteristics of immune tolerance lend support to research findings suggesting that dying microglia with lost Aβ phagocytic function may contribute to amyloid plaque growth, further exacerbating the disease process [88].

Multiple studies have reported alterations in glucose metabolism in microglia within various AD mouse models. For instance, both APP-PS1 [89–91] and 3xTg AD mice [92, 93] and exhibit increased glycolysis and upregulation of glycolytic enzymes, although these effects diminish with age [92]. Similarly, microglia from 5 × FAD mice display elevated levels of free NAD(P)H, indicating enhanced glycolytic activity [94]. However, it is essential to consider the broader metabolic landscape, as parallel pathways—such as the pentose phosphate pathway (PPP)—also contribute to NAD(P)H levels [95, 96]. In response to immune activation by lipopolysaccharide (LPS) and interferon-γ (IFN-γ), microglia shift their glucose metabolism toward both glycolysis and the PPP [97]. The PPP, which begins with glucose-6-phosphate, serves as a key metabolic pathway operating alongside glycolysis [96]. It provides essential cellular components such as NADPH, ribose-5-phosphate, and glycolytic intermediates. NADPH, in particular, is critical for regulating oxidative stress and maintaining reactive oxygen species (ROS) homeostasis [98, 99]. Its levels are primarily controlled by glucose-6-phosphate dehydrogenase (G6PD), the rate-limiting enzyme of the PPP [100, 101]. A delicate balance in G6PD activity is essential, as dysregulation can lead to oxidative stress-induced cellular damage [101, 102]. In microglia exposed to Aβ, increased glucose consumption is accompanied by upregulated G6PD activity, leading to elevated mitochondrial ROS production and pro-inflammatory metabolic activation [103, 104]. NADPH also plays a significant role in lipid metabolism, as it is required for fatty acid synthesis [98, 105]. This underscores the intricate interconnectivity of metabolic pathways in microglia, demonstrating that glucose metabolism does not function in isolation but is closely coordinated with other metabolic processes [106, 107]. Understanding these interactions is crucial for elucidating microglial metabolic phenotypes in AD.

In addition to glucose, microglia can utilize lactate and pyruvate, key intermediates of glucose metabolism, as alternative energy sources [59, 107–109]. Upon exposure to inflammatory stimuli such as LPS or Aβ, microglia undergo a metabolic shift from oxidative phosphorylation (OXPHOS) to glycolysis, leading to increased lactate production [46, 110]. While this shift initially supports an acute immune response, excessive lactate accumulation may also influence inflammation. Some studies suggest that exogenous lactate can mitigate LPS-induced inflammatory responses in both BV2 microglial cell lines and mice, indicating potential anti-inflammatory effects [111, 112]. However, other investigations have shown that lactate exposure impairs microglial phagocytic activity, reducing their ability to engulf and clear Aβ aggregates [113]. Enhanced glycolysis in microglia can also lead to a self-reinforcing metabolic cycle, further amplifying glycolytic activity. This phenomenon is particularly evident in 5 × FAD mice, where microglia surrounding Aβ plaques exhibit a sustained glycolytic state [114]. A major contributing factor is lactate-induced histone lactylation, an epigenetic modification that enhances the expression of glycolysis-related genes, thereby reinforcing metabolic reprogramming [114, 115]. This positive-feedback loop disrupts microglial homeostasis, intensifies neuroinflammation, and ultimately accelerates AD progression. Interestingly, targeted inhibition of PKM2 (a key glycolytic enzyme that promotes lactate production) in microglia of 5XFAD mice resulted in a reduction of amyloid plaques and improvements in cognitive function [114]. These findings suggest that glycolytic reprogramming and lactate metabolism play a crucial role in microglial-mediated neuroinflammation. However, further research is needed to fully elucidate the mechanistic link between lactate-driven epigenetic changes and microglial dysfunction in AD.

Microglia utilize glucose through various glucose transporters (GLUTs), with GLUT3 being the predominant isoform under normal physiological conditions. However, in inflammatory environments, GLUT1 expression is upregulated to facilitate increased glucose uptake and enhance glycolysis, thereby meeting the heightened energy demands associated with immune activation [116–118]. In AD, cerebral glucose metabolism declines significantly, a phenomenon strongly linked to cognitive impairment [119, 120]. Studies have reported reduced levels of GLUT1 in the brains of AD patients [121] and AD mouse models [122, 123]. This reduction in GLUT1 expression has been associated with exacerbated amyloid pathology and worsened cognitive function. Notably, in APP-PS1 model mice, GLUT1 levels in the hippocampus decline significantly only after extensive amyloid deposition [123], while cortical GLUT1 expression progressively decreases with aging in Tg2576 mice [124]. However, However, these studies did not specify the exact cellular contributors (e.g., neurons, microglia, or astrocytes) to the observed decrease in GLUT1 expression. While impaired glucose metabolism in neurons is well recognized as a driver of neurodegeneration [125, 126], the specific changes in microglial glucose metabolism and their contributions to AD progression remain less understood. Recent studies integrating 2-deoxy-2-[18F]fluoro-D-glucose positron emission tomography (FDG-PET) with single-cell RNA sequencing (scRNA-seq) have revealed a strong association between increased glucose uptake and metabolic reprogramming of microglia in the hippocampus of 5XFAD mice [127]. scRNA-seq analysis has identified distinct microglial subpopulations with unique glucose metabolism signatures, including a subset characterized by elevated GLUT1 expression, enhanced glycolysis, and reduced OXPHOS, which became more prevalent as AD pathology progressed [127]. These findings suggest that GLUT1 upregulation in microglia may serve as an adaptive response to sustain energy production in response to the disease. As AD progresses, glucose metabolism within the CNS becomes increasingly heterogeneous across different cell types [128–131]. Microglia exhibit diverse metabolic profiles depending on their activation states, which may also correlate with their proximity to amyloid plaques [94, 132]. A separate FDG-PET study using an AD mouse model demonstrated that microglia exhibit higher glucose uptake than neurons and astrocytes [133]. Additionally, research utilizing TSPO-PET (18-kDa translocator protein PET) has established a positive correlation between microglial activation and glucose uptake in both AD and tauopathy mouse models, as well as in human patients [133]. These findings emphasize the importance of distinguishing between impaired neuronal glucose metabolism and microglial metabolic adaptation to accurately determine their respective contributions to AD pathology. Immune cells, including microglia, rely on glycolysis and mitochondrial metabolism to meet their energy and biosynthetic demands [134, 135].

Glycogen, a branched polymer of glucose, serves as a cellular energy reserve and a source of biosynthetic precursors [136]. While glycogen is primarily stored in the liver and muscles to provide energy when needed [137, 138], its role in the CNS has remained underexplored due to technical limitations in measuring glycogen levels [139]. This knowledge gap is particularly pronounced in microglia. However, recent technological advancements have enabled researchers to uncover novel insights into microglial glycogen metabolism [139].

Prostaglandin E2 (PGE2) is a bioactive lipid mediator that exerts its effects through four distinct G protein-coupled receptors (EP1–EP4), among which EP2 plays a critical role in inflammatory signaling and metabolic regulation [140, 141]. Recent studies in aged microglia have shown that activation of the PGE2-EP2 signaling pathway promotes glycogen synthesis, leading to glucose sequestration and reduced metabolic flux through glycolysis and mitochondrial respiration [142]. Interestingly, inhibition of the PGE2-EP2 pathway has been found to decrease glycogen accumulation, enhance glycolysis, and restore spatial memory and inflammatory responses in aged mice [142]. Glycogen accumulation has also been identified in specific microglial subpopulations [143, 144]. Among these, dark microglia (DM) represent a newly identified phenotype enriched under conditions of chronic stress, aging, and AD pathology [143, 145]. First described by Bisht et al., dark microglia exhibit ultrastructural features indicative of oxidative stress, including dense cytoplasm, nucleoplasmic condensation, dilated endoplasmic reticulum (ER), and mitochondrial abnormalities [143, 146]. Further studies by St-Pierre et al. identified glycogen granules as a unique feature distinguishing dark microglia from other microglial phenotypes [144]. Notably, glycogen-rich dark microglia are predominantly located near Aβ plaques and dystrophic neurites in the hippocampus of APP-PS1 mice [144]. The presence of glycogen granules in these microglia suggests a potential metabolic shift toward glycolysis in response to Aβ plaque-induced stress. However, under pathological conditions, glycogen accumulation may also reflect impaired glucose metabolism, possibly due to decreased glucose availability. This metabolic dysfunction may compromise the microglial response to stressors such as Aβ, ultimately influencing AD progression. Given the emerging role of glycogen metabolism in microglial function, further research is warranted to elucidate its relationship with glucose metabolism and its broader impact on AD pathogenesis.

A comprehensive understanding of glucose metabolism in AD requires an in-depth investigation of microglial metabolic profiles. However, current single-cell RNA sequencing (scRNA-seq) data are limited, as they are typically derived from a small number of mice and restricted to specific brain regions. This constraint hampers the ability to construct a holistic view of glucose metabolism across the entire brain in AD. To overcome this limitation, future scRNA-seq analyses should incorporate a broader dataset that accounts for key factors such as the spatial heterogeneity of microglia [147] and their proximity to amyloid plaques. These refinements will provide a more precise characterization of microglial glucose metabolism in AD and offer deeper insights into how metabolic alterations contribute to disease progression.

#### Lipid metabolism

In addition to glucose, lipids serve as another crucial energy source influencing microglial activation. These lipids play essential roles in regulating membrane structure and cellular signaling within the brain. Microglia, equipped with diverse receptors, monitor the brain's microenvironment, with lipid rafts—sphingolipid- and cholesterol-rich microdomains in the plasma membrane—playing a significant role in processes such as receptor trafficking and signaling cascades [148, 149]. On the other hand, intracellular lipids, including those in mitochondria and signaling molecules, can modulate immune responses by affecting metabolic pathways [150]. Increasing attention is being directed toward the role of microglia-mediated lipid and lipoprotein metabolism in the brain and its implications for systemic and neuroinflammatory diseases [62]. The rapid growth of scRNA-seq studies has revealed a wide range of microglial phenotypes, far beyond the classical M1/M2 types [151–153]. These studies have highlighted changes in gene expression related to lipid and lipoprotein metabolism and their association with various microglial phenotypes [154]. Moreover, accumulating genetic and biological evidence underscores the significant role of lipid metabolism in AD. Over a century ago, Alois Alzheimer described 'lipid granules' in AD brains [155], raising early suspicions about the potential role of disrupted lipid metabolism in the disease. These suspicions have since been reinforced by genome-wide association studies (GWAS), which have identified several lipid-related genes, including APOE, APOJ, and ABCA7, as strong AD risk factors [13, 15, 156]. Recent comprehensive single-cell transcriptomic analyses, including scRNA-seq and single-nucleus RNA sequencing (snRNA-seq), have further defined the transcriptional identities of microglia with disease-specific states [157–160].

For instance, Keren-Shaul et al. identified a unique population of microglia, termed disease-associated microglia (DAM), in AD mouse model [157]. Key genes upregulated in DAM, such as *APOE, TREM2, LPL,* and *CST7* are involved in lipid metabolism and phagocytosis [157, 161–163]. This transcriptional profile suggests that, in the later stages of AD, microglia may shift toward lipid metabolism as a primary energy source for Aβ clearance. However, findings from DAM in mouse models may not fully capture the complexity of microglial states in human AD [164–166]. Recent snRNA-seq analyses by Sun et al. have expanded this understanding by revealing a greater diversity of microglial transcriptional states in human AD brains compared to the DAM signatures observed in AD mouse models [160]. These microglial substates were functionally distinct, characterized by features such as ribosome biogenesis, inflammatory responses, and lipid processing. A distinct microglial state linked to cholesterol and lipid homeostasis—characterized by genes such as *PPARG*,* APOE*,* ABCA1*, and *TREM2*—was significantly enriched in AD [160]. This microglial state showed a strong correlation with amyloid plaques, tau tangles, Braak staging, and cognitive decline. Furthermore, state- and stage-specific differential analyses revealed that inflammatory responses precede lipid regulation in microglia during disease progression [160]. These findings underscore the strong link between inflammation and lipid metabolism, emphasizing the critical role of microglia in AD pathogenesis.

Marschallinger et al. discovered a novel microglia subpopulation, lipid-droplet-accumulating microglia (LDAM) in the aging brain [65]. These microglia exhibit defective phagocytosis, excessive reactive oxygen species (ROS) production, and elevated pro-inflammatory cytokine secretion [65, 167]. LDAM exhibit a distinct transcriptome signature, setting them apart from previously described microglia states observed in aging and neurodegeneration, such as DAM and neurodegenerative microglia (MGnD). LDAM were also identified near amyloid plaques in 5XFAD mice[66]and in chimaeric human–mouse AD models [158]. Similar cells have been observed in hippocampal tissue from AD patients [66], suggesting that LDAM may play a significant role in the progression of AD and other neurodegenerative disorders. These findings highlight how lipid dysregulation in microglia may contribute to neurodegeneration.

Lipid metabolism, encompassing processes such as lipid uptake, lipolysis, fatty acid beta oxidation (FAO), lipogenesis, and fatty acid synthesis, provides essential energy for microglial activation [62]. Microglia express a wide range of genes related to lipid metabolism, including those involved in FAO [29, 168], a process crucial for their metabolic function. During FAO, fatty acids (FAs) are liberated from lipid droplets (LDs) and transported into mitochondria, where they fuel OXPHOS, generating the energy needed for cellular activities [169, 170]. This pathway becomes particularly important when glucose availability is limited, as demonstrated in studies on macrophages, which share many functional similarities with microglia [171, 172]. In energy-stressed environments, FAs serve as a vital alternative energy source, enabling microglia to maintain their essential functions in the CNS [173, 174]. Changes in FAO can affect microglial energy production and phenotype, potentially influencing their response to AD pathology [175, 176].

Lipoprotein lipase (LPL), a DAM-associated gene and an identified AD risk factor [157, 177], plays a crucial role in lipid metabolism in microglia, regulating lipid droplet accumulation and FAO [178, 179]. Loss of LPL in microglia leads to increased pro-inflammatory lipid profiles, impaired lipid uptake, and reduced FAO [178], contributing to neuroinflammation and microglial dysfunction. In AD, this impaired LPL function disrupts lipid homeostasis, facilitating the accumulation of neurofibrillary tangles and amyloid plaques [62, 177, 180]. Notably, in response to Aβ exposure, microglia upregulate LPL expression, which enhances their phagocytosis of Aβ [75, 181]. Additionally, in an AD mouse model, pharmacological inhibition of hexokinase 2 (HK2), a key glycolytic enzyme, increased LPL expression, which in turn regulated lipid metabolism and was associated with reduced amyloid load and improved cognitive function [75]. Similarly, ABCA7, another LOAD risk gene involved in lipid transport, is critical for maintaining phospholipid and cholesterol homeostasis [182, 183]. Genetic studies have linked ABCA7 loss-of-function variants to an increased risk of AD [184–187], yet the precise mechanisms underlying this association remain incompletely understood. Recent evidence suggests that ABCA7 is involved in the crosstalk between lipid metabolism and neuroinflammation. Metabolomic network analysis identified glycerophospholipid metabolism, linoleic acid metabolism, and α-linolenic acid metabolism as key pathways affected by ABCA7 haploinsufficiency following acute inflammatory stimulation [188]. This was accompanied by increased levels of eicosapentaenoic acid, oleic acid, and palmitic acid, further supporting ABCA7’s role in coordinating lipid metabolism and immune responses [189]. In addition, a metabolome-wide association study (MWAS) has identified a link between ABCA7 and peripheral blood lactosylceramide (LacCers) levels, suggesting a potential role in sphingolipid metabolism [189]. Further analysis in ABCA7 knockout mice revealed decreased LacCer levels in the brain, while overall sphingolipids, ceramides, and hexosylceramides were increased, indicating that ABCA7 may be required for proper processing of sphingolipids into LacCer species [189]. These findings suggest that ABCA7 deficiency alters ceramide metabolism, potentially contributing to microglial lipid imbalance and neuroinflammation.

ABCA7-deficient microglia exhibit reduced Aβ phagocytic clearance, which aligns with the increased Aβ accumulation observed in the brains of ABCA7-deficient mice [190, 191]. Additionally, the rare ABCA7 p.A696S variant has been associated with exacerbated amyloid burden and plaque-associated neuritic dystrophy in 5xFAD mice, along with reduced amyloid-induced microglial activation and altered brain lipid profiles, including increased lysophosphatidylethanolamine (LysoPE) levels [192]. Transcriptomic analysis of ABCA7-A696S knock-in 5XFAD mice further revealed increased expression of DAM genes including *APOE*, *TREM2*, and *AXL* [192]. These findings suggest that ABCA7 dysfunction may disrupt microglial lipid metabolism and immune regulation, thereby contributing to AD pathogenesis by impairing Aβ clearance and promoting neuroinflammation.

Microglia accumulate LDs when exposed to Aβ, particularly near amyloid plaques in AD patients and mouse models [66, 193]. This lipid accumulation impairs microglial Aβ clearance [66, 193]. Lipidomic analyses have shown a significant decrease in free fatty acids (FFAs) and an increase in triacylglycerols (TAGs) as the key metabolic transition underlying LD formation in microglia exposed to Aβ [66]. This shift in lipid composition is mediated by DGAT2, which converts FFA to TAG [194, 195]. Increased levels of DGAT2 are found in microglia from AD brains [66]. Interestingly, the inhibition of DGAT2 has been shown to improve microglial uptake of Aβ, suggesting a potential therapeutic strategy for reducing amyloid burden in AD [66]. Shippy et al. recently conducted a comprehensive analysis of lipid metabolism-related gene expression changes in microglia under AD and neuroinflammatory conditions [176]. Their microarray analysis of 5XFAD mice revealed numerous differentially expressed genes (DEGs) related to lipid metabolism compared to wild-type mice. Functional enrichment and network analyses identified several biological processes and molecular functions associated with these DEGs, including cholesterol homeostasis (e.g., *HMGCR, ANGPTL3, APOE, INSIG1, LPL*), fatty acid metabolic/biosynthetic process (e.g., *ANGPTL3*, *ACADS*, *ACACA, FABP3*), and triglyceride metabolism *(*e.g., *HMGCR, ANGPTL3, APOC4, APOE, LPL)* [176]. Furthermore, recent study has demonstrated that reducing LD accumulation in microglia restores Aβ phagocytosis and leads to a reduction in amyloid plaque deposition, highlighting the therapeutic potential of targeting microglial lipid metabolism in AD [193].

Building on these findings, recent studies have further elucidated the molecular mechanisms that link microglial lipid metabolism to AD pathology, identifying key signaling pathways involved in this process. Notably, two recent studies have highlighted the roles of the integrated stress response (ISR) [196] and REV-ERBα signaling [197] in mediating microglial lipid dysregulation and dysfunction. ISR activation in microglia, observed in both AD patients and mouse models, promotes the secretion of toxic lipids and impairs neuronal survival. This response exacerbates synapse loss and tau pathology, positioning ISR-activated microglia as a critical neurodegenerative phenotype [196]. Similarly, REV-ERBα, known for regulating lipid metabolism in the periphery, also modulates lipid metabolism and inflammatory responses in microglia within the CNS. Microglial REV-ERBα deletion leads to excessive lipid droplet accumulation, impaired tau clearance, and exacerbated neuroinflammation in tauopathy model [197]. This effect was observed specifically in male mice, highlighting the potential sex-dependent role of REV-ERBα in microglial lipid metabolism and tau pathology [197]. Together, these recent findings emphasize how specific signaling pathways mediate the link between lipid metabolism dysregulation and microglial dysfunction, offering potential avenues for therapeutic intervention in AD.

Emerging evidence suggests that, beyond the role of lipid metabolism in microglial dysfunction, pathogenic tau directly disrupts lipid metabolism, further exacerbating neurodegeneration. Olesova et al. demonstrated significant alterations in mitochondrial-associated lipid metabolism during various stages of tau pathology in a transgenic rat model (SHR-24) expressing human truncated tau protein [47]. Their analysis revealed that tau accumulation disrupts metabolic pathways, including FAO, TCA cycle, and creatine metabolism, contributing to mitochondrial dysfunction. In vitro studies further showed that truncated tau increases LD formation in microglial mitochondria, reducing ATP production and exacerbating oxidative stress [47]. Similarly, in PS19 mice expressing human P301S mutant tau, microglia enriched in LDs displayed elevated oxidative stress and an inflammatory phenotype, impairing tau clearance and driving neurodegeneration [198]. These findings highlight how lipid dysregulation and LD accumulation disrupt mitochondrial function and microglial activity, creating a vicious cycle that amplifies both amyloid and tau pathologies.

Additionally, tau-induced changes in cellular membrane fluidity and lipid order further disrupt microglial function [47]. Alterations in lipid composition can significantly affect receptor expression and receptor-mediated signaling pathways, modulating cell polarization and activity [61, 149, 199, 200]. In microglia, tau has been shown to decrease membrane lipid order, shifting it toward a more liquid phase, potentially contributing to energy imbalance and impaired microglial responses [47]. These findings indicate that tau pathology may disrupt lipid metabolism and energy homeostasis in microglia, potentially contributing to neurodegenerative processes.

A recent study by Li et al. identified a potential mechanism underlying excessive LD accumulation in microglia in tauopathy models [198]. The researchers found that tauopathy neurons release excess unsaturated lipids, which are then taken up by nearby microglia, leading to significant LD accumulation in these cells [198]. Notably, LD accumulation in microglia appears to be associated with the exacerbation of tau pathology [201]. Importantly, the extent of LD buildup in microglia has been found to correlate with hippocampal neuron loss [198], suggesting a potential association between lipid dysregulation and neurodegeneration in tauopathies.

#### Amino acids metabolism

Microglia demonstrate remarkable metabolic flexibility in their energy acquisition. When glucose availability is limited, they can efficiently shift their metabolism to utilize amino acids as an alternative fuel, highlighting their bioenergetic adaptability [30]. Beyond their role in protein synthesis, amino acids can be catabolized through various pathways to generate ATP, providing an alternative energy source when needed [202]. For instance, glutamine act as a crucial bioenergetic substrate for microglia under hypoglycemic conditions [30]. Glutamine, the most abundant free amino acid in the brain, is converted into glutamate by glutaminase and then further transformed into α-ketoglutarate by glutamate dehydrogenase, enabling it to enter the TCA cycle to support mitochondrial metabolism for the production of energy [203]. However, imbalances in glutamine metabolism during microglial inflammatory activation can potentially lead to detrimental outcomes [204–206]. Glutamine metabolism, particularly through the enzyme glutaminase (GLS), appears to regulate microglial activation and pro-inflammatory responses [207]. Abnormal elevation of the GLS isoform glutaminase C (GAC), the rate-limiting enzyme in glutaminolysis, has been observed in activated microglia and early AD mouse brain tissues [208]. Gao et al. demonstrated that GAC upregulation in microglia enhances the release of pro-exosome, potentially exacerbating neuroinflammation in AD [208]. Therefore, dysregulation of glutamine utilization may contribute to chronic neuroinflammation and microglial dysfunction in AD pathogenesis.

The APOE4 allele is well known as the greatest genetic risk factor for LOAD. Microglia expressing APOE4 exhibit exaggerated immune responses and a diminished capacity to clear Aβ compared to microglia expressing APOE2 or APOE3 [209, 210]. These functional differences underscore the impact of APOE genotype on microglial behavior in the context of AD. Notably, a significant increase in GLS activity was observed in hippocampal microglia from APOE4 knock-in mice compared to age-matched wild-type controls [69]. Chronic administration of JHU-083, a glutaminase antagonist, improved cognition and attenuated the rise of GLS activity in APOE4 knock-in mice [69]. Collectively, these studies indicate that while glutamine is a vital metabolic substrate for microglia, its excessive availability or altered metabolism during pro-inflammatory responses can potentially contribute to neuroinflammation and neurotoxicity.

Alongside glutamine, other amino acids, including tryptophan and arginine, also play crucial roles in microglial metabolism and immune responses [211, 212]. Tryptophan serves as a precursor for neuroactive compounds and influences microglial function, contributing to either neurotoxic or neuroprotective effects [213]. The majority of tryptophan is metabolized via the kynurenine pathway, and alterations in this metabolic pathway have been linked to various human diseases, including psychiatric disorders, autoimmunity, neurodegeneration, and AD [51, 214–217]. The rate-limiting enzyme in the kynurenine pathway, indoleamine 2,3-dioxygenase (IDO), is upregulated in the AD brain and promotes the conversion of tryptophan into quinolinic acid (QA) [215, 218]. This pathway is particularly active in microglia exposed to Aβ, leading to an increase in QA production. Elevated IDO levels in AD are associated with neurofibrillary tangles and Aβ plaques, suggesting a potential role of tryptophan metabolism in AD pathology [215, 218, 219]. Additionally, QA, a key metabolite of tryptophan degradation, has been implicated in neurotoxicity in various inflammatory brain disorders, including AD [215, 220, 221]. During neuroinflammation, microglial activation promotes the shift in kynurenine metabolism towards increased QA production [222, 223]. QA is secreted from microglia and acts as an NMDA receptor agonist, inducing neurotoxicity [224]. Furthermore, studies have shown that QA treatment induces tau phosphorylation in neurons [225].

Arginine is metabolized through two competing pathways: one via nitric oxide synthase (iNOS) to generate nitric oxide (NO) and citrulline, and the other via arginase to produce urea and ornithine [226]. iNOS is a marker of the pro-inflammatory M1 microglial phenotype, while arginase-1 is associated with the anti-inflammatory M2 phenotype [227–229]. NO, synthesized through iNOS, inhibits Complex IV of the mitochondrial electron transport chain, disrupting mitochondrial function and shifting microglial metabolism toward glycolysis [230]. NO produced by iNOS, upregulated in microglia upon Aβ exposure, has been shown to induce neurotoxicity [231–233]. Independent studies also report increased tau phosphorylation in neurons exposed to NO [234], suggesting that NO produced via iNOS-mediated arginine metabolism contributes to AD progression by driving inflammation and potentially influencing tau phosphorylation in neurons. Conversely, M2 microglia express arginase-1, which catalyzes the conversion of arginine into polyamine precursors, promoting anti-inflammatory processes [228]. In the hippocampal region of APP-PS1 mice, Arginase-1 + microglia, which contain more Aβ than iNOS + microglia, were upregulated and involved in Aβ clearance [235].

In summary, amino acid metabolism plays a pivotal role in regulating microglial functional and phenotypic transitions in both in vivo and in vitro contexts. Among key metabolic pathways, tryptophan metabolism through the kynurenine pathway, mediated by IDO, and arginine metabolism via iNOS and arginase-1, serve as crucial links between microglial activation and AD pathology. However, the precise molecular mechanisms that connect these metabolic reprogramming events to neuroinflammation and Aβ/tau pathology remain incompletely understood. Furthermore, while glutamine metabolism has been extensively investigated, the roles of other amino acids, such as tryptophan and arginine, remain relatively underexplored, highlighting the need for further research to fully elucidate their contributions to AD pathogenesis.

### Molecular mediators of microglial immunometabolism in AD

#### TREM2

TREM2 is a membrane protein predominantly expressed on microglia in the CNS [35, 161, 236], playing critical roles in the wide-ranging microglial functions, including proliferation, differentiation, phagocytosis, metabolism, survival, lipid handling and inflammatory responses [38, 40, 237–240]. Given its essential role in microglial function and immune homeostasis, genetic variants of TREM2 that lead to loss of function are strongly associated with an increased risk of developing AD [241–243]. Recent studies emphasize that TREM2, which is upregulated in DAM [154, 157], facilitates the transition of microglia to the DAM phenotype [157, 244, 245] while modulating metabolism to support microglial activation and Aβ clearance [35, 39, 157, 245–247]. Research using knockout models has demonstrated that TREM2 activates a metabolic program involving glycolysis, the PPP, and the TCA cycle to support phagocytosis [35]. Glucose uptake, the critical energy source for this metabolic pathway, depends on TREM2 and varies with microglial activation status [133]. Microglia exhibit distinct transcriptomic and metabolic profiles depending on their level of TREM2 expression, with these differences becoming more pronounced near amyloid plaques [248]. TREM2 deficiency in microglia from 5XFAD mice leads to a downregulation of glucose transporters and glycolytic enzymes. This energy deficit, induced by TREM2 deficiency, activates AMPK and impairs mTOR signaling, resulting in mitochondrial damage and increased autophagy [35]. These autophagy-related changes align with the multi-omics analyses in patients with amyloid pathology and AD-like brain organoid models [249], suggesting that alterations in autophagy-related pathways, which are central regulators of cellular metabolism, may be key contributors to AD pathogenesis.

Similar to TREM2-deficient microglia, microglia expressing the R47H variant of TREM2 exhibit metabolic impairments, including reduced mitochondrial oxidative phosphorylation and an inability to undergo glycolytic reprogramming. These metabolic deficits compromise energy production, phagocytosis, and adaptive responses to inflammation, impairing their ability to clear Aβ42 and further emphasizing the critical role of TREM2 in maintaining microglial metabolic fitness [250]. Likewise, alterations in microglial metabolic pathways have been discovered in the brain of AD patients carrying the R47H variant [251]. Piers et al. found that the observed deficits in microglial metabolic regulation and associated functions in TREM2 variants are caused by dysregulation of the PPARγ/p38MAPK signaling pathway [250]. They showed that by activating these pathways, the metabolic deficits of microglia can be improved, leading to the restoration of Aβ phagocytosis.

TREM2 also plays a crucial role in regulating microglial cholesterol metabolism, particularly during chronic phagocytic challenges. In microglia lacking the TREM2, there is a disruption in the regulation of genes involved in lipid metabolism, resulting in an accumulation of cholesterol ester (CE) and impaired transport of cholesterol within the brain [239, 252]. This indicates that TREM2 is a vital component in maintaining optimal lipid homeostasis in microglia. Knockdown of TREM2 results in increased lipid synthesis and decreased cholesterol clearance and lipid hydrolysis, further impacting microglial phenotypes. The critical role of TREM2 in regulating cholesterol transport and metabolism in microglia, largely identified through knockout studies, was further corroborated by recent research demonstrating an inverse relationship between TREM2 expression and free cholesterol levels [248]. Microglia expressing high levels of TREM2 showed significantly lower levels of free cholesterol and palmitoylcarnitine compared to their low TREM2 expressing microglia [248], suggesting high lipid catabolism and cholesterol efflux in TREM2-high microglia. Overall, elevated TREM2 expression in microglia correlates with enhanced energetic and metabolic capacities, as well as improved cholesterol management. This suggests a pivotal role for TREM2 in optimizing microglial function through metabolic regulation.

#### APOE

Given that TREM2 is a key regulator of microglial lipid metabolism, APOE, another major player in lipid handling, also significantly influences microglial immunometabolism. Upregulation of microglial APOE is a significant feature observed in both AD patients [164, 253] and AD mouse brains [157, 253–255]. A comprehensive multi-omics study revealed that APOE4 significantly alters microglial metabolism and immune responses [256]. APOE4 microglia, isolated from APOE4 KI mice, exhibit increased aerobic glycolysis with elevated HIF-1α expression and a disrupted TCA cycle compared with APOE3 microglia [70]. These microglia show increased APOE expression and upregulation of genes involved in glucose metabolism, lipid processing, and pro-inflammatory cytokines [70]. These findings are partially consistent with another work that reported that APOE4 significantly impairs the metabolic function of human iPSC-derived microglia, inhibiting both glycolysis and OXPHOS, which in turn hinders their phagocytosis, migration, and overall metabolic activity, while simultaneously exacerbating cytokine secretion [257, 258]. Furthermore, APOE4 5XFAD mice exhibited enriched genes associated with the DAM/MGnD signature in the cortex and hippocampus compared to APOE3 5XFAD mice, indicating an induced microglial transcriptomic switch in response to amyloid pathology [70]. In addition to its effects on glucose metabolism, APOE4 also disrupts lipid homeostasis in microglia, leading to lipid droplet accumulation and impaired lipid processing, particularly in the context of amyloid pathology [256]. Interestingly, deletion of APOE4 in microglia restored the MGnD response to chronic neurodegeneration and promoted neuroprotection by alleviating tau and amyloid pathology [256].

In addition to DAM/MGnD microglia, a recent study identified a novel population of microglia, termed terminally inflammatory microglia (TIM) [259]. TIM, characterized by the expression of inflammatory signals and stress markers, increases with age and APOE4 presence, showing reduced cellular energetics (including depleted citric acid cycle, glycolysis, and sugar metabolism pathways), features of ROS detoxification, and altered amino acid metabolism [259]. An analogous population has been identified in the human AD brain. TIM exhibits impaired Aβ clearance and altered cell–cell communication during aducanumab treatment [259], suggesting that it may contribute to AD risk and pathology, particularly in APOE4 carriers and the elderly, making it a potential therapeutic target.

The brain's primary lipid transporter, APOE, has also been demonstrated to exert isoform-specific effects on lipid and cholesterol metabolism. Microglia derived from human induced pluripotent stem cells (iPSCs) expressing APOE4 were found to contain more LDs [73], supporting recent findings that APOE4 in microglia influences lipid metabolism and potentially contributes to AD pathology. In this recent study by Haney et al. snRNA-seq analysis of AD brain tissue revealed a population of LD-enriched microglia characterized by high ACSL1 expression, an enzyme involved in LD formation [260]. This ACSL1-positive LDAM population was most abundant in patients with the APOE4/4 genotype. In iPSC-derived microglia, Aβ upregulated ACSL1 expression, resulting in triglyceride synthesis and LD accumulation in an APOE-dependent manner. Notably, secreted factors from these LD-laden microglia led to tau phosphorylation and neurotoxicity in an APOE-dependent manner. Recently, the impact of APOE isoform-dependent microglial changes on AD pathogenesis has been investigated using a human microglia xenotransplantation model [261]. In this study, ATAC-seq and RNA-seq were used to distinguish the effects of various APOE isoforms on microglia in AD from epigenomic and transcriptomic perspectives. Significant transcriptomic and epigenetic differences were found in microglia depending on the APOE isoform. APOE4 was shown to potentially increase AD risk by upregulating genes related to lipid accumulation and impairing microglial proliferation, migration, and immune responses [261].

#### TREM2-APOE interaction

TREM2 and APOE are pivotal in AD pathogenesis due to their interconnected roles in lipid metabolism, microglial activation, and Aβ clearance [262, 263]. While TREM2 facilitates phagocytosis by recognizing lipid ligands [161, 264], APOE transports lipids and cholesterol, interacting with microglial receptors involved in lipoprotein metabolism [163]. TREM2 and APOE collaboratively regulate microglial responses to Aβ pathology [159, 262, 263], with TREM2 promoting phagocytosis and APOE facilitating lipid handling. This activation drives the MGnD transition by promoting metabolic and inflammatory reprogramming in microglia, a process accompanied by TGF-β1 suppression [159]. Transcriptomic analyses indicate that TREM2-APOE signaling promotes MGnD-like microglia while downregulating homeostatic genes such as P2ry12, Tmem119, and Tgfbr1, suggesting that TGF-β1 suppression may be necessary for MGnD activation, yet its complete loss could impair microglial homeostasis and cognitive function [265].

Recent findings highlight that APOE4 disrupts this balance, leading to excessive lipid droplet accumulation in microglia, particularly in APP-PS1:APOE4 KI models [256]. This metabolic dysregulation mirrors that of TREM2-deficient microglia, where disrupted lipid metabolism and TGF-β1 signaling jointly promote a pro-inflammatory state [266]. TREM2 deficiency disrupts cholesterol homeostasis, leading to cholesteryl ester accumulation and lipid droplet formation, which shifts microglia toward a pro-inflammatory phenotype [266]. Notably, blocking APOE4-TGF-β1 signaling restores MGnD-like phenotypes and enhances Aβ clearance in AD models, highlighting the dual role of TGF-β1 in microglial function—maintaining homeostasis under physiological conditions but potentially impeding MGnD activation and Aβ clearance in AD pathology—thereby underscoring its complex interplay with the TREM2-APOE axis.

Given its crucial role in regulating lipid metabolism and immune function, TREM2 acts as a key receptor for APOE [263, 267, 268], integrating metabolic and inflammatory responses to maintain microglial function in AD pathology. This interaction is essential for coordinating microglial responses to Aβ pathology, further emphasizing its central role in AD progression [12, 269]. Indeed, transcriptomic studies in 5XFAD mice have identified TREM2 and APOE as two of the most upregulated genes in microglia actively engaged in amyloid phagocytosis, underscoring their importance in microglial activation and function in AD pathology [255, 270].

Importantly, TREM2 and APOE are critical for the formation of a microglial barrier surrounding plaques, a structural response that limits amyloid spread and associated toxicity. In the absence of either gene, this protective microglial clustering around Aβ plaques is significantly impaired, highlighting their cooperative role in modulating plaque-associated microglial activity [271]. Microglia expressing APOE4 exhibit a reduced ability to respond to Aβ compared to those expressing APOE3. This deficit is further exacerbated in the absence of TREM2, leading to a significantly diminished capacity for Aβ phagocytosis [272]. In AD, the TREM2-APOE axis plays a crucial role in microglial activation and Aβ clearance, a function that extends to other neurodegenerative diseases where impaired microglial responses contribute to pathology [159]. A recent study has revealed that externalized phosphatidylserine (ePtdSer), generated from nutrient-deprived neurons surrounding Aβ plaques, serves as the driving force behind TREM2-dependent Aβ phagocytosis in microglia [273]. Intriguingly, microglia expressing APOE4 demonstrated reduced TREM2-dependent Aβ phagocytosis via ePtdSer compared to those expressing APOE3 [273].

While the role of TREM2-APOE interactions in Aβ pathology is relatively well-characterized, their contribution to tau pathology remains less understood, reflecting the broader complexity of AD progression. Recent evidence suggests that APOE4 serves as the primary driver of dysfunction in tau-related pathology [274]. Interestingly, the impact of TREM2 deficiency on tau pathology is dependent on APOE isoform, revealing a more nuanced interplay between these two factors in AD. In TREM2-deficient P301S tauopathy mice expressing murine APOE, neuroinflammation is reduced, and brain atrophy is mitigated, suggesting a neuroprotective role of TREM2 deficiency [275]. However, in P301S mice expressing human APOE4, TREM2 deficiency exacerbates tau pathology and neurodegeneration, despite a reduction in TREM2-dependent microgliosis [274]. Notably, human APOE4 promotes abnormal lipid accumulation in microglial lysosomes, independent of TREM2, further reinforcing its central role in driving tau-related pathology. These findings highlight that the interplay between TREM2 and APOE extends beyond ligand binding, actively shaping microglial responses to neurodegeneration. Given the therapeutic interest in TREM2 modulation, isoform-specific investigations into APOE-TREM2 interactions will be crucial for refining treatment strategies. Furthermore, addressing the interplay of these mechanisms in both amyloid and tau pathologies will be essential for developing comprehensive and effective therapeutic approaches.

APOE-TREM2 interactions are modulated by APOE isoform and lipidation status [263, 268, 276, 277], which may in turn affect microglial function in AD pathology. The degree of APOE lipidation differs among isoforms, with APOE2 exhibiting the highest level and APOE4 showing the lowest [278–281]. TREM2 preferentially binds to non-lipidated APOE, and APOE4 generally displays a higher binding affinity than APOE2 [276, 277], possibly due to its lower lipidation level. Differences in APOE lipidation across isoforms influence not only TREM2 interactions but also key microglial functions, such as Aβ clearance, lipid homeostasis, and inflammatory responses [282].

A recent study by Guo et al. reported that lipidated APOE2 exhibits reduced LDLR binding, which appears to be a protective mechanism in AD by limiting excessive toxic lipid uptake and preventing microglial overactivation [283]. These findings suggest that APOE lipidation may play a key role in modulating interactions with various APOE receptors, which could be closely linked to the progression of AD pathology.

Phospholipid composition also varies in an APOE isoform-specific manner, contributing to differences in lipidation [272, 284] and microglial responses to Aβ [272]. APOE3 lipoproteins, compared to APOE4, enhance microglial migration, promote Aβ uptake, and induce a stronger activation response, with these effects being more pronounced in the absence of TREM2 [272]. These findings suggest that isoform-specific variations in phospholipid composition contribute to differences in APOE lipidation, which may, in turn, influence APOE-TREM2 interactions and microglial function in Aβ pathology [272].

Lipidated APOE plays a crucial role in Aβ metabolism by forming complexes with Aβ, facilitating its rapid clearance by microglia, and reducing Aβ toxicity [278, 285]. However, poorly lipidated APOE4 exhibits reduced binding affinity to Aβ, leading to slower clearance, increased Aβ toxicity, and heightened inflammation [210, 285–287]. Furthermore, the R47H variant of TREM2 not only shows reduced binding affinity for APOE [263, 267, 277] but also disrupts downstream microglial signaling pathways [288, 289], impairing Aβ clearance and exacerbating neuroinflammation. Aβ interacts with both the lipid-binding and receptor-binding domains of APOE, further complicating APOE-TREM2 interactions and their consequences for microglial function [290, 291].

Given these complexities, a comprehensive understanding of the interactions between APOE and its various receptors, including but not limited to TREM2, is crucial for elucidating the mechanisms underlying microglial dysfunction in AD. In particular, research should focus on how APOE isoform differences, lipidation status, and phospholipid composition influence these receptor interactions and downstream signaling pathways. Gaining these insights may facilitate the development of targeted therapeutic strategies that effectively address both Aβ and tau pathologies in AD.

#### Hypoxia-inducible factors

Metabolic plasticity refers to the ability of cells to adapt their metabolic processes in response to changing environmental conditions or internal needs. The drivers of metabolic reprogramming that have been most extensively studied are changes in nutrient availability and oxygen levels [292, 293]. Hypoxia occurs when cells, especially during inflammation, experience increased oxygen consumption [294, 295]. It is a well-established driver of glycolysis, as oxygen deficiency restricts OXPHOS efficiency. Hypoxia-inducible factor 1 (HIF-1) is a heterodimeric transcription factor consisting of HIF-1α and HIF-1β that plays a crucial role in cellular response to low oxygen conditions, or hypoxia [295, 296]. Under hypoxic conditions, HIF-1 facilitates sustained ATP production by upregulating hexokinase and phosphofructokinase to enhance anaerobic glycolysis and by increasing GLUT1 expression to enhance glucose uptake [295, 297, 298]. HIF-1α stabilization is not limited to hypoxia but can also be stabilized under normoxic conditions via pro-inflammatory cytokines (e.g., TNF-α and IL-1β) [299], metabolic intermediates of the TCA cycle (e.g., succinate and fumarate) [300], and NO [301, 302]. These factors inhibit prolyl hydroxylase (PHD) activity, preventing HIF-1α degradation [300, 301]. Such normoxic regulatory mechanisms, observed in macrophages [297, 303], may also influence microglial metabolism.

Hypoxia and inflammation are closely linked, with HIF-1 serving as a key switch in glycolytic reprogramming and acting as a crucial transcriptional regulator of immunity and inflammation [294, 295, 298, 304]. Baik et al. demonstrated that primary microglia exposed to Aβ induce metabolic reprogramming from OXPHOS to glycolysis via the mTOR-HIF-1α pathway, leading to an acute inflammatory state [46]. However, chronic Aβ exposure results in metabolic deficits and downregulation of the mTOR-HIF-1α pathway, impairing microglial function and reducing their phagocytic capacity [46]. Restoration of this pathway via IFN-γ treatment in 5xFAD mice reactivated microglial immune responses and enhanced phagocytic function, suggesting metabolic modulation as a potential therapeutic approach for AD [46]. Conversely, Yang et al. proposed a contrasting strategy that targets the same pathway but employs an opposing approach [305]. They developed an immunometabolic reprogramming nanomodulator (GAF NPs) that inhibits the mTOR-HIF-1α pathway, shifting microglial metabolism from glycolysis to OXPHOS and promoting an M2 anti-inflammatory phenotype. This inhibition was accompanied by GLUT1 suppression, reducing glucose uptake and further limiting glycolytic activity. By dampening excessive inflammatory responses, this strategy led to cognitive improvement and decreased Aβ accumulation in APP-PS1 mice [305]. A recent study introduced a peptide-drug conjugates (PDCs)-based nanoplatform designed to enhance microglial function by restoring the mTOR-HIF-1α pathway, particularly in later stages of AD pathology [306]. Notably, this study used the same APP-PS1 model as Yang et al., yet adopted a strategy similar to Baik et al. by upregulating HIF-1α. The researchers found that the activation state of the mTOR-HIF-1α pathway was highly dependent on disease progression. In APP-PS1 mice, HIF-1α and p-mTOR were significantly elevated at 7 months of age, aligning with the timing of Yang et al.'s intervention, which aimed to suppress this pathway. However, by 9 months of age, a sharp decline in HIF-1α and mTOR activity was observed, indicative of an immune-suppressed state resembling the chronic phase described by Baik et al. In this context, PDCs treatment successfully restored mTOR-HIF-1α signaling, leading to improved microglial function and neuroprotection in advanced-stage AD [306]. These findings reinforce the notion that disease progression and microglial metabolic states can vary depending on the animal model and experimental time points. The opposing strategies proposed by Baik et al. and Yang et al. may not be mutually exclusive but rather context-dependent, influenced by the stage of AD pathology at which the intervention is applied. Therefore, the therapeutic success of targeting the HIF-1α-mTOR pathway may depend on precise timing to maximize its efficacy, underscoring the importance of understanding microglial metabolic transitions across different disease stages for the development of effective immunometabolic interventions in AD.

In 5XFAD mice, microglia exhibit distinct transcriptional profiles depending on whether they contain Aβ [307]. In 5XFAD mice, microglia containing Aβ exhibited a distinct transcriptional profile characterized by an active HIF-1α regulon. Notably, microglia with this transcriptional signature are also increased in postmortem AD brains [307]. This upregulation of HIF-1α may support the enhanced metabolic and phagocytic activities required to manage the pathological environment induced by Aβ plaques in AD. Similar findings have been observed in Aβ-associated microglia of APP-PS1 mice, where activation of the HIF-1 pathway and transcription of mitochondrial-related genes were noted. This was accompanied by mitochondrial elongation, an adaptive response to support aerobic respiration under low nutrient and oxygen conditions [308]. However, sustained HIF-1 activation or prolonged hypoxia can induce microglial quiescence, diminishing their responsiveness to Aβ. This low-reactivity state diminishes microglial clustering around Aβ plaques, ultimately exacerbating Aβ pathology [308].

Additionally, the complement C3a receptor (C3aR) signaling pathway has been implicated in microglial metabolic regulation, in part through its influence on HIF-1α activity [309]. Under hypoxia-mimicking conditions, C3aR-deficient microglia exhibited reduced HIF-1α levels, which was accompanied by the downregulation of its glycolysis-promoting target genes, including *PDK1*, *PFKL*, and *PFK2* [309]. Notably, these microglia exhibited resistance to hypoxia mimetic-induced metabolic changes and lipid droplet accumulation. Consistent with these findings, C3aR expression is elevated around Aβ plaques in APP-KI mice, where microglia with high C3aR expression show metabolic impairments characterized by increased HIF-1α signaling and disrupted lipid metabolism, ultimately affecting their response to Aβ pathology [309]. However, C3aR deficiency in these mice restored lipid profiles, reduced lipid droplet accumulation, and enhanced microglial phagocytic capacity. These changes were accompanied by a decrease in Aβ burden and a modest improvement in cognitive function [309]. This suggests that the C3aR/HIF-1α axis plays a critical role in regulating microglial metabolic adaptation and may be a potential therapeutic target in AD.

Further evidence linking HIF-1α to microglial metabolism comes from studies implicating key AD risk genes, such as TREM2 and APOE4, in its regulation. In an AD mouse model, TREM2 deficiency leads to significant metabolic alterations in microglia, including reduced mitochondrial mass and downregulated HIF-1α expression [35]. This suggests that TREM2 plays a vital role in maintaining microglial metabolic fitness through regulation of HIF-1α-mediated glycolytic pathways. Conversely, APOE4 promotes a glycolytic phenotype in microglia via increased HIF-1α activation, particularly in aging. Aged APOE4 microglia exhibit elevated aerobic glycolysis, accumulation of glycolytic metabolites, and the emergence of a DAM-like microglial subpopulation [70]. This HIF-1α-high, DAM-like phenotype is linked to disrupted cholesterol metabolism, further highlighting the interplay between APOE4, microglial metabolic states, and AD pathology [70].

These findings underscore the complex and context-dependent role of HIF-1α in AD pathology. The effects of HIF-1α modulation likely depend on microglial activation states, disease progression, and cellular metabolism, necessitating a nuanced approach when considering it as a therapeutic target.

#### AMPK

Adenosine monophosphate-activated protein kinase (AMPK), a serine/threonine protein kinase, serves as an energy sensor and master regulator, playing a pivotal role in maintaining cellular energy homeostasis [310]. Beyond its role as an energy sensor, AMPK coordinates multiple metabolic pathways, including glucose metabolism, lipid metabolism, and protein synthesis, to adapt cellular function under energy stress conditions [310–312]. Classical activation of AMPK occurs when the ratio of adenine nucleotides (AMP to ATP and/or ADP to ATP) within the cell increases. Upon activation, AMPK restores ATP levels during metabolic stress by simultaneously inhibiting ATP-consuming biosynthetic pathways and activating ATP-generating catabolic pathways [78]. Furthermore, AMPK phosphorylates multiple transcription factors such as FOXO3, CREB, and p300 that play crucial roles in biosynthetic pathways and metabolism regulation [310]. Through these mechanisms, AMPK rapidly restores energy balance and transcriptionally modulates cell metabolism in response to long-term energy deficits [313].

Autophagy plays a crucial role in maintaining microglial homeostasis by enabling cells to adapt to metabolic stress through the degradation and recycling of cellular components [314]. AMPK activation promotes autophagy by inhibiting mTOR, a key regulator of cell growth that suppresses autophagy in nutrient-rich conditions [315]. This AMPK-mTOR balance is particularly crucial in microglial function, as it regulates inflammatory responses and microglial polarization [316–318]. In neurodegenerative diseases such as AD, persistent metabolic stress and inflammation impair autophagic flux, leading to the accumulation of dysfunctional proteins and organelles [314, 319]. By modulating catabolic and anabolic processes [320], AMPK signaling contributes to maintaining cellular adaptation and homeostasis, potentially influencing Aβ clearance and neuroinflammation dynamics.

As AMPK activation exerts anti-inflammatory effects [321], metabolic changes in response to immune stimuli may suppress AMPK function, leading to immune responses such as pro-inflammatory cytokine secretion and inflammasome activation. As late-stage AD progresses, chronic low-level inflammation persists, exacerbating neurotoxicity. Aβ-driven neuroinflammation is partly mediated through the NLRP3 inflammasome [322], which is activated in microglia following AMPK inhibition and Syk signaling activation. Research shows that AMPK deactivation leads to metabolic dysregulation, mitochondrial fragmentation, and reactive oxygen species production, thereby promoting NLRP3 inflammasome activation [323]. As a central regulator of cellular metabolism, AMPK influences multiple metabolic pathways, including TCA cycle, FAO, cholesterol homeostasis, and glycolysis, to maintain energy balance. As these metabolic processes are also involved in NLRP3 inflammasome activation [206, 324–326], AMPK dysfunction may further exacerbate neuroinflammatory reponses in AD by disrupting metabolic homeostasis. In this context, NSAIDs or metformin, which have a direct anti-inflammatory function and enhance AMPK activity, may serve as potential adjuvant treatments to modulate microglial immunometabolism [323, 327–330].

Interestingly, INPP5D, a microglial gene identified through GWAS as a late-onset AD risk factor [13, 67, 331], has been implicated in autophagy and NLRP3 inflammasome activation [332], both of which are closely linked to AMPK signaling and metabolic regulation [333, 334]. While INPP5D expression is elevated in amyloid plaque-associated microglia [332, 335], its functional role appears to be context-dependent, with potential contributions to both protective and pathological processes [336–338]. A recent study reported that the functional decline caused by INPP5D reduction is associated with disrupted autophagy flux and increased activation of the NLRP3 inflammasome [332]. However, INPP5D can modulate the downstream activity of multiple receptors involved in microglial activation and function [337], and AMPK signaling can influence intracellular pathways that are also affected by INPP5D, necessitating further research to clarify its role in AD pathogenesis.

AMPK plays a central role in regulating cellular metabolism, influencing multiple pathways including glucose metabolism, lipid homeostasis, mitochondrial function, energy production and utilization, and protein synthesis [311]. Through these metabolic networks, AMPK modulates cellular stress responses, coordinating autophagy, mitophagy, and lysosomal function while also shaping inflammatory pathways such as NLRP3 inflammasome activation [318]. Given its ability to integrate metabolic and immune signaling, AMPK represents a key node linking energy homeostasis to neuroinflammatory regulation in AD pathogenesis.

These multifaceted roles of AMPK in regulating microglial inflammatory responses offer insights into its function and impact on AD pathogenesis. As a central metabolic regulator, AMPK influences various processes, including autophagy, mitochondrial homeostasis, and immune responses, making it a compelling target for therapeutic intervention [339–342]. However, AMPK operates within a complex signaling network involving LOAD risk genes such as TREM2 and other metabolic regulators [343, 344], meaning its modulation could have varying effects depending on cellular context, disease stage, and microglial activation states [57, 341]. Directly targeting AMPK may lead to unintended consequences, highlighting the need to carefully consider whether modulating upstream regulators or downstream pathways—such as autophagy and NLRP3 inflammasome activation—offers a more viable therapeutic strategy for AD.

#### Hexokinase

Hexokinases (HKs) are the initial rate-limiting enzymes in glucose metabolism, catalyzing the phosphorylation of glucose to glucose-6-phosphate (G-6-P), thereby initiating the first committed step in glycolysis. Recent studies on tissue-resident macrophages, including microglia, have highlighted the critical role of hexokinase in glycolysis and its influence on mitochondrial function, which is dynamically regulated by inflammatory activation and plays a key role in immune responses [345–347]. Mammals express five isoforms of hexokinase (HK), with the four primary isoforms being HK1, HK2, HK3, and HK4 (also known as glucokinase), along with an additional isoform, HKDC1. Among these, HK1, HK2, and HK4 are localized to the outer mitochondrial membrane (OMM), where they interact with voltage-dependent anion channels (VDACs) to facilitate ATP-dependent glucose phosphorylation [348]. In the brain, HK1, HK2, and HK3 are expressed, but notably, HK2 levels were significantly elevated in AD patients [50, 75, 349]. RNA-seq analyses across various brain cell types, supported by in situ hybridization and immunoblotting, revealed that HK2 is selectively enriched in microglia, both in murine and human brains, compared to other brain cells. Moreover, HK2 expression was markedly elevated in microglia undergoing immune activation or associated with disease states [349]. Surprisingly, contrary to the expectation that depleting HK2—the initial enzyme in energy metabolism—would suppress metabolism, its deletion instead increased microglial energy demand and enhanced phagocytic activity [75, 349]. These findings suggest that HK2 overexpression in microglia does not efficiently support the energy requirements for phagocytosis. Interestingly, under chronic conditions such as AD, genetic or pharmacological depletion of HK2 induced a metabolic shift in microglia from glucose-dependent metabolism to lipid utilization. This metabolic adaptation enhanced ATP production, thereby delaying the onset of AD symptoms in mouse models [75, 350]. Beyond its role in glycolysis, HK2 also has non-metabolic functions [351–353], contributing to microglial inflammatory responses. Its impact on disease progression appears to be gene-dosage-dependent, as complete deletion of HK2 exacerbates inflammation and mitochondrial dysfunction, whereas partial reduction (haploinsufficiency) alleviates AD pathogenesis in AD model mice [50].

These findings suggest that the balance between HK2's metabolic and non-metabolic functions is tightly regulated in response to physiological and pathological stimuli. Further research into the multifaceted roles of HK2 will be essential to understanding the reciprocal regulation of metabolic and inflammatory processes in microglia and their contributions to AD pathogenesis and progression.

## Conclusion and future directions

Recent advances in multi-omics technologies, including single-cell genomics, transcriptomics, proteomics, metabolomics, and epigenomics, have unveiled the remarkable heterogeneity of microglial states under both physiological and pathological conditions. This diversity reflects the dynamic nature of microglia, which continuously adapt to genetic and environmental influences throughout aging and neurodegenerative processes (Fig. 1, Table 1). While this complexity poses challenges for drug development, it also presents new opportunities for therapeutic intervention in Alzheimer’s disease (AD). Given the critical role of metabolic regulation in microglial function, targeting metabolic pathways has emerged as a promising therapeutic approach for AD [43, 45, 92, 305]. However, the intricate interplay between microglial transcriptional states, metabolic adaptations, and functional roles necessitates a more refined therapeutic strategy. The identification of distinct microglial subpopulations, each exhibiting unique metabolic profiles and functional states [354–356], highlights the need for a comprehensive and context-dependent understanding of microglial behavior in AD. Despite these insights, methodological limitations—such as incomplete capture of microglial states and insufficient spatial resolution in pathology assessment—continue to impede progress.

A recent study has provided a detailed gene expression map across multiple brain regions in both AD patients and healthy individuals [357]. This study identified cell type- and region-specific genetic alterations in AD, revealing transcriptomic differences associated with diverse pathological factors [357]. These findings underscore the complexity of microglial metabolic networks and emphasize the necessity of considering genetic predispositions, environmental influences, and comorbidities in the development of targeted therapeutic strategies.

Growing evidence suggests a bidirectional relationship between AD pathology and microglial metabolic dysfunction. Aβ and tau aggregates drive metabolic reprogramming, impairing microglial immune function [46, 53, 86, 87, 358]. Conversely, pre-existing metabolic disturbances may predispose microglia to a dysfunctional state, further exacerbating AD progression [60, 359, 360]. This interplay may differ between sporadic and familial AD, where familial AD is primarily driven by early Aβ accumulation, whereas sporadic AD appears to be more influenced by systemic metabolic dysfunction and neuroinflammation [361–363]. Moreover, metabolic disorders such as obesity and type 2 diabetes, which are closely linked to an increased risk of AD [364–369], along with their associated metabolic hormones, may influence brain immunity and microglial function [370–374]. Individuals with these metabolic conditions may exhibit reduced microglial resilience to pathological stimuli such as Aβ, thereby impairing their ability to mount an effective response. This diminished adaptive capacity may lead to defective immune responses and compromised Aβ clearance, ultimately accelerating AD progression.

Moving forward, research efforts should aim to clarify the mechanistic links between metabolic pathways and microglial dysfunction, as well as to identify novel therapeutic targets that restore microglial homeostasis. A deeper understanding of how systemic metabolic status influences microglial function will be essential for designing effective AD treatments. Additionally, integrating these findings within the spatial and temporal framework of disease progression is crucial for the development of precision-targeted therapies. Addressing these challenges may pave the way for metabolic-based therapeutic interventions, offering a transformative approach to AD treatment.

## Abbreviations

Aβ: Amyloid-beta
AD: Alzheimer’s disease
AMPK: Adenosine monophosphate-activated protein kinase
APOE: Apolipoprotein E
APOE4: APOE-ε4
CE: Cholesterol ester
DAMs: Disease-associated microglia
FAO: Fatty acid oxidation
FAs: Fatty acids
FFAs: Free fatty acids
G-6-P: Glucose-6-phosphate
GAC: Glutaminase C
GLS: Glutaminase
GLUTs: Glucose transporters
HIF-1: Hypoxia-inducible factor 1
HK2: Hexokinase II
iMGL: iPSC-derived microglia
iPSCs: Induced pluripotent stem cells
LD: Lipid droplet
LDAM: Lipid-droplet-accumulating microglia
LDs: Lipid droplets
LPL: Lipoprotein Lipase
MGnD: Microglial neurodegenerative phenotype
OXPHOS: Oxidative phosphorylation
PPP: Pentose phosphate pathway
ROS: Reactive oxygen species
scRNA-seq: Single-cell RNA sequencing
TAGs: Triacylglycerols
TCA: Tricarboxylic acid
TIM: Termed late-stage inflammatory microglia
TREM2: Triggering receptor expressed on myeloid cells 2
